# Three New Cases of Melioidosis, Guadeloupe, French West Indies

**DOI:** 10.3201/eid2603.190718

**Published:** 2020-03

**Authors:** Bénédicte Melot, Sylvaine Bastian, Nathalie Dournon, Eric Valade, Olivier Gorgé, Anne Le Fleche, Charlotte Idier, Mireille Vernier, Elisabeth Fernandes, Bruno Hoen, Sébastien Breurec, Michel Carles

**Affiliations:** University Hospital of Guadeloupe, Pointe-à-Pitre, France (B. Melot, S. Bastian, N. Dournon, C. Idier, B. Hoen, S. Breurec, M. Carles);; Ecole du Val-de-Grâce, Paris, France (E. Valade);; Aix Marseille University, Marseille, France (E. Valade, O. Gorgé); Institut Pasteur, Paris (A. Le Fleche);; Hospital of Basse-Terre, Basse-Terre, France (M. Vernier, E. Fernandes);; Pasteur Institute of Guadeloupe, Pointe-à-Pitre (S. Breurec);; University of the French West Indies and French Guiana, Pointe-à-Pitre (B. Hoen, S. Breurec, M. Carles)

**Keywords:** melioidosis, *Burkholderia pseudomallei*, endemicity, Caribbean, Guadeloupe, French West Indies, bacteria, France

## Abstract

Melioidosis has been detected in the Caribbean, and an increasing number of cases has been reported in the past few decades, but only 2 cases were reported in Guadeloupe during the past 20 years. We describe 3 more cases that occurred during 2016–2017 and examine arguments for increasing endemicity.

Melioidosis, caused by the telluric gram-negative rod *Burkholderia pseudomallei*, is endemic in Southeast Asia and northern Australia (*1*) but may be underdiagnosed in other tropical regions (*2*). Increasing occurrences have been reported in the Caribbean during the past few decades among persons with no exposure to known endemic areas (*3*–*5*). Tropical environmental conditions and the presence of this bacterium in soil samples in the Caribbean support the plausibility of endemicity (*3*). We describe 3 new cases detected in Guadeloupe during 2016–2017.

Patient 1 was a 54-year-old man, receiving renal replacement therapy, with a history of hypertensive vascular nephropathy. He developed a pulmonary form of melioidosis in November 2016. Thoracoabdominal computed tomography (CT) scan showed bilateral nodular lesions. *B. pseudomallei* grew from bronchoalveolar lavage fluid obtained by fiberoptic bronchoscopy. Treatment with ceftazidime (6 g/d intravenously) was given for 2 weeks and switched to trimethoprim/sulfamethoxazole (TMP/SMX) (320/1,600 mg 2×/d orally) for 1 month, then changed to doxycycline because rash developed. The patient complied poorly with treatment; he died in March 2017 under unknown circumstances.

Patient 2 was a 66-year-old woman with a history of arterial hypertension and diabetes mellitus, a subcutaneous abscess in the prepubic area surgically treated without microbiological identification (June 2016), a lumbar hematoma (March 2017), and bacteremic obstructive pyelonephritis caused by *Escherichia coli* (April 2017). In April 2017, she developed a severe and disseminated form of melioidosis with pneumonia, bacteremia, and deep abscess. CT scan showed multiple pulmonary nodes consistent with hematogenous pneumonia, a deep abscess between kidney and psoas, and splenic emboli. *B. pseudomallei* was isolated from blood cultures performed at admission and from the abscess. The patient developed multiple complications: acute respiratory distress syndrome, systemic candidiasis, renal failure, hemodynamic failure, nonspecific encephalopathy, refractory septic shock related to catheter infection, and bacteremia caused by extended spectrum β-lactamase *Klebsiella pneumoniae.* In the intensive care unit, she was treated with ceftazidime (6 g/d for 24 d), then with meropenem (1 g 3×/d) plus TMP/SMX (320/1,600 mg 2×/d). Blood cultures grew *B. pseudomallei* until day 40. The patient died on day 60 from multiple organ failure.

Patient 3 was a 52-year-old man with a history of chronic alcoholism. He developed pneumonia in April 2017. Thoracic tomography showed an excavated condensation of the right middle lobe, right lower infiltrates, and multiple right hilar nodes. Bronchoalveolar lavage fluid contained *B. pseudomallei*. Intravenous ceftazidime (2 g 3×/d) for 40 days followed by oral TMP/SMX (320/1,600 mg 2×/d) slowly improved the clinical status, but 1 month after starting oral antimicrobial drug therapy, he had a drug reaction that caused eosinophilia and systematic symptoms. He died a year later despite appropriate treatment.

All patients were born and had always lived in the western part of Guadeloupe and Les Saintes islands, the rainiest places in Guadeloupe (1,500–5,500 mm of rainfall per year in 2017 [Météo France, http://www.meteofrance.gp/climat/pluies-annuelles/rr_an_guadeloupe]). The patients reported no travel history to endemic countries. All had a history of potential occupational or recreational exposure to *B. pseudomallei* (as farmers, gardeners) and predisposing risk factors, such as diabetes mellitus, chronic renal diseases, and alcoholism (*1*). The clinical manifestations of disease were classical, but all patients experienced severe side effects during their treatments, and the mortality rate was 100% (Table), which is much higher than in most series of reported cases, underlining the severity of this disease. 

**Table:** Main comparative data for 3 cases of melioidosis in Guadeloupe, French West Indes*

Characteristic	Patient 1	Patient 2	Patient 3
Age, y	54	66	52
Sex	M	F	M
Place of birth	Guadeloupe	Guadeloupe	Guadeloupe
Place of residence	Bouillante	Deshaies	Les Saintes
Rainfall, mm/y	2,500–3,000	1,500–2,000	1,500–2,000
Concurrent conditions	Chronic renal failure (vascular nephropathy)	Diabetes	Chronic alcohol intake
Possible means of inoculation	Gardening without gloves	Animals breeding, gardening without gloves	Animals breeding, gardening without shoes
Clinical presentation according to the Infectious Disease Association of Thailand†	1: Multifocal infection with bacteremia (45% of cases, 87% mortality)	3: Localized infection (42% of cases, 9% mortality)	3: Localized infection (42% of cases, 9% mortality)
Time from first clinical signs to death	11 mo	2 mo	16 mo
Organ involvement	Pneumonia	Disseminated (psoas abscess, lung abscesses, bacteremia)	Pneumonia
MLST	ST92	ST95	ST92
Treatment	Ceftazidime + TMP/SMX, then doxycycline; TMP/SMX discontinued due to rash	Ceftazidime, meropenem, TMP/SMX	Ceftazidime + TMP/SMX; TMP/SMX discontinued due to DRESS syndrome
Outcome	Death	Death	Death

*DRESS, drug reaction with eosinophilia and systematic symptoms; MLST, multilocus sequence typing; ST, sequence type; TMP/SMX, trimethoprim/sulfamethoxazole. †Punyagupta S. Melioidosis: review of 686 cases and presentation of a new clinical classification. In: Punyagupta S, Sirisanthana T, Stapatayavong B, eds. Melioidosis. Bangkok: Bangkok Medical; 1989:217–29; Leelarasamee A, Bovornkitti S. Melioidosis: review and update. Rev Infect Dis. 1989; 11:413–25.

These 3 cases of melioidosis were identified over a 6-month period, in contrast with only 2 cases diagnosed and reported during the previous 20 years in Guadeloupe. The identification of the isolate from the first case was performed locally by the API-20NE system (bioMérieux, https://www.biomerieux.com) and confirmed by matrix-assisted laser desorption/ionization time-of-flight (MALDI-TOF) mass spectrometry and by real-time PCR (*6*) at a reference laboratory in France*.* The isolates from the other cases were not identified correctly by the API-20NE system, as often described (*7*). However, after the first case, we were aware that a wrinkled colony-forming, oxidase-positive, gram-negative bacillus resistant to colistin and aminoglycosides could be *B. pseudomallei.* Thus, the strains were sent to the reference laboratory for confirmation. All the isolates were genotyped by multilocus sequence typing (*8*). They belonged to sequence type (ST) 92 (n = 2) and 95 (n = 1), 2 clones previously described in Central and South America and Caribbean islands: Brazil (ST92), Puerto Rico (ST95), Martinique, and Mexico (ST92 and ST95 in both areas) (*9*). This finding highlights the potential role of this region as a reservoir for these clones.

Our experience suggests that the incidence of *B. pseudomallei* infection is probably underestimated in the Caribbean because of inadequate diagnostic laboratory facilities and the lack of knowledge about melioidosis among physicians and microbiologists. The tropical climate in this region provides suitable conditions for bacterial survival, and of elevated alcoholism and diabetes rates among Caribbean populations cause weakened immunity that could lead to increased infection risk (*10*). Therefore, investigation of soil samples should be undertaken to identify the most likely sources of human infection in this area.
